# EZH2 Down-Regulation Exacerbates Lipid Accumulation and Inflammation in *in Vitro* and *in Vivo* NAFLD

**DOI:** 10.3390/ijms141224154

**Published:** 2013-12-12

**Authors:** Serena Vella, Daniela Gnani, Annalisa Crudele, Sara Ceccarelli, Cristiano De Stefanis, Stefania Gaspari, Valerio Nobili, Franco Locatelli, Victor E Marquez, Rossella Rota, Anna Alisi

**Affiliations:** 1 Department of Oncohematology, Bambino Gesù Children’s Hospital, IRCCS, Rome 00165, Italy; E-Mails: serena.vella@opbg.net (S.V.); stefania.gaspari@opbg.net (S.G.); franco.locatelli@opbg.net (F.L.); 2 Liver Research Unit, Bambino Gesù Children’s Hospital, IRCCS, Rome 00165, Italy; E-Mails: daniela.gnani@yahoo.it (D.G.); annalisa.crudele@gmail.com (A.C.); sara.ceccarelli@opbg.net (S.C.); cristiano.destefanis@opbg.net (C.D.S.); 3 Hepato-Metabolic Disease Unit, Bambino Gesù Children’s Hospital, IRCCS, Rome 00165, Italy; E-Mail: nobili66@yahoo.it; 4 Department of Pediatric Hematology-Oncology, University of Pavia, Pavia 27100, Italy; 5 Chemical Biology Laboratory, Frederick National Laboratory for Cancer Research, CCR, National Cancer Institute, NIH, Frederick, MD 21702, USA; E-Mail: marquezv@mail.nih.gov

**Keywords:** NAFLD, EZH2, DZNep, microRNAs

## Abstract

Non-alcoholic fatty liver disease (NAFLD) is one of the most prevalent, chronic liver diseases, worldwide. It is a multifactorial disease caused by complex interactions between genetic, epigenetic and environmental factors. Recently, several microRNAs, some of which epigenetically regulated, have been found to be up- and/or down-regulated during NAFLD development. However, in NAFLD, the essential role of the Polycomb Group protein Enhancer of Zeste Homolog 2 (EZH2), which controls the epigenetic silencing of specific genes and/or microRNAs by trimethylating Lys27 on histone H3, still remains unknown. In this study, we demonstrate that the nuclear expression/activity of the EZH2 protein is down-regulated both in livers from NAFLD rats and in the free fatty acid-treated HepG2. The drop in EZH2 is inversely correlated with: (i) lipid accumulation; (ii) the expression of pro-inflammatory markers including TNF-α and TGF-β; and (iii) the expression of miR-200b and miR-155. Consistently, the pharmacological inhibition of EZH2 by 3-Deazaneplanocin A (DZNep) significantly reduces EZH2 expression/activity, while it increases lipid accumulation, inflammatory molecules and microRNAs. In conclusion, the results of this study suggest that the defective activity of EZH2 can enhance the NAFLD development by favouring steatosis and the de-repression of the inflammatory genes and that of specific microRNAs.

## 1. Introduction

Over the past two decades, the incidence of non-alcoholic fatty liver disease (NAFLD) has risen sharply in several Western Countries and across Asia, while the prevalence of this disease in children is of growing concern [1–3]. The term NAFLD implies a variety of liver histopathological disorders resulting from the effect of the interactions between specific genetic backgrounds (*i.e*., the presence of specific gene polymorphisms) and environmental factors (hypercaloric diets and/or sedentary lifestyle) [4–6]. Besides these pathogenetic factors, it is now widely accepted that epigenetic mechanisms may also contribute to the development and progression of NAFLD causing the more severe, non-alcoholic steatohepatitis (NASH) [7,8].

Epigenetic mechanisms comprise processes including DNA methylation, nucleosome repositioning and regulation of non-coding small RNAs, such as microRNAs (miRNAs) [9–11]. The epigenetic control of DNA accessibility and the consequent regulation of gene-expression also depend on histone modifications, such as acetylation, methylation and phosphorylation of their lysine rich amino-terminal “tails” [12]. The methyltransferase Polycomb Group (PcG) protein Enhancer of Zeste Homolog 2 (EZH2), which is the catalytic factor of the Polycomb Repressor Complex 2 (PCR2), regulates gene expression by methylating Lysine 27 on histone H3 (H3K27me3) resulting in the transcriptional repression of specific genes during embryogenesis and tumorigenesis [13,14]. EZH2-mediated epigenetic control, known for protein-coding genes, also occurs on the miRNA loci, as demonstrated in many tumours [15]. EZH2 up-regulation was frequently detected in hepatocellular carcinoma (HCC) suggesting its potential role as a sensitive malignancy marker in this tumour. There are also reports demonstrating the crucial role of EZH2 in multiple tumours [16–18].

Interestingly, the epigenetic processes are affected by the genetic makeup and environmental factors, including diet. In fact, excessive nutrient intake may precipitate dynamic epigenetic modifications in some specific genes causing an individual to become prone to diet-related diseases, such as metabolic syndrome and NAFLD [19,20]. Unfortunately, apart from the most extensively studied miRNAs, the role of the other epigenetic mechanisms during NAFLD pathogenesis still remains little understood [7,8,21]. Regulation by DNA methylation has been demonstrated in humans for genes encoding proteins involved in insulin resistance and inflammation [22]. Further, the development of liver steatosis is associated with several epigenetic abnormalities in mice [22,23]. Recently, Pirola *et al*. [24] demonstrated that the cytosine methylation of the hepatic mitochondrial DNA is associated with the histological severity of NAFLD.

Although these findings suggest that epigenomic control may be crucial for the development and progression of NAFLD, there is a distinct lack of research demonstrating the potential role of EZH2 in NAFLD pathogenesis. Therefore, in this study we have investigated the EZH2 expression and activity in *in vivo* and *in vitro* NAFLD and its potential correlation with disease features.

## 2. Results and Discussion

### 2.1. EZH2 Expression and Activity in *in Vivo* and *in Vitro* NAFLD

**Figure 1.**
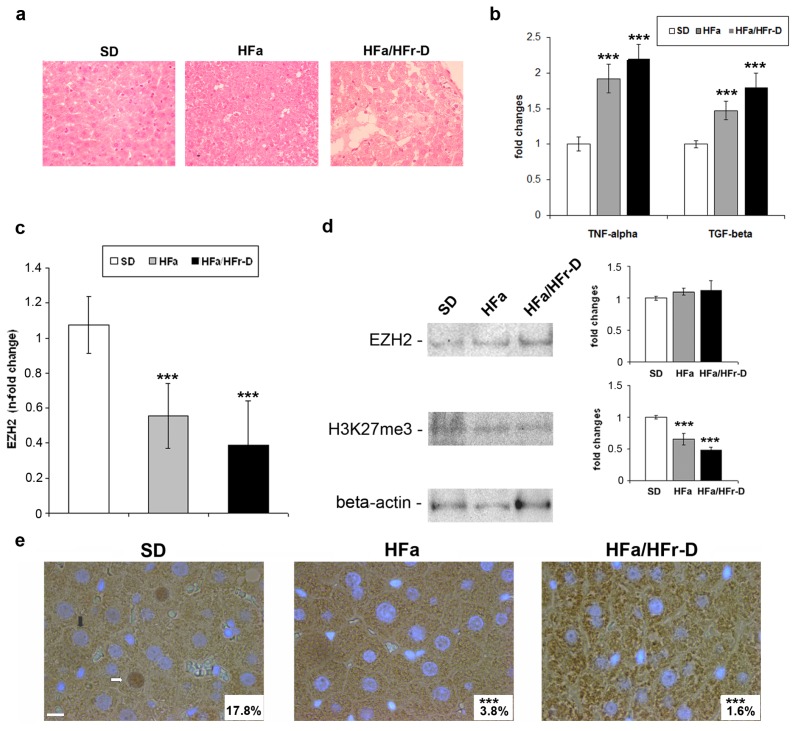
(**a**) Hematoxylin-Eosin staining of the livers from SD, HFa and HFa/HFr-D rats (Magnification 400×); (**b**) Plasma protein levels of TNF-α and TGF-β in SD, HFa and HFa/HFr-D rats are reported as fold inductions. ***p < 0.001 *vs*. SD group; (**c**) EZH2 mRNA expression levels in the SD, HFa and HFa/HFr-D rats. The histograms represent the mean ± SD of at least two independent experiments in triplicate. ***p < 0.001 *vs*. control rats; (**d**) Representative western blotting of EZH2, H3K27me3 and β-actin in total liver lysates from the SD, HFa and HFa/HFr-D rats. Quantitative bars and the corresponding error bars (SD) are referred to the mean of three independent experimental determinations with respect to β-actin considered as 1. ***p < 0.001 *vs*. SD group; and (**e**) Representative immunohistochemical staining for EZH2 in the liver tissues from the SD, HFa and HFa/HFr-D rats. Nuclei are counterstained with 4′,6-diamidino-2-phenylindole (DAPI). Black and white arrows indicate the EZH2-negative and EZH2-positive nuclei, respectively. The lower, right-hand squares in white reveal the mean percentage of the EZH2-positive nuclei (Magnification 600×). ***p < 0.001 *vs*. control. Scale bar, 10 μm.

As previously reported [25], the high-fat (HFa) and high-fat/high-fructose diet fed (HFa/HFr-D) rats metabolically and histologically resemble human NAFLD. In Figure 1a the Hematoxylin-Eosin (H-E) staining of the liver tissues from rats fed on standard-diet (SD), HFa or HFa/HFr-D, is reported. In particular, the livers from the HFa rats displayed a pattern of micro- and macro-vacuolar steatosis, while those from the HFa/HFr-D rats exhibited more features of human NAFLD, including liver steatosis, ballooning, inflammation and fibrosis. The intra-hepatic expression of the pro-inflammatory and pro-fibrogenic molecules such as TNF (tumour necrosis factor)-α and TGF (tumour growth factor)-β are undetectable by mRNA and/or protein analysis as they are quickly processed in the liver before being released into the bloodstream. Therefore, in our models, we evaluated the various diet-related changes in these cytokines by assessing their plasma levels. The quantitative ELISA method revealed a statistically significant progressive increase in the circulating levels of TNF-α and TGF-β from SD to HFa, as well as from HFa to HFa/HFr-D rats (Figure 1b). These data confirm that the high-fat and high-fructose dietary habits induce not only a histological pattern in rats, but also a systemic inflammation profile that mimics the NAFLD in humans. Moreover, a combined diet (HFa/HFr-D) leads to the more severe form of the disease.

In this work, we also investigated for the first time, the expression and the potential function of EZH2 in the *in vivo* and *in vitro* NAFLD models. First, the hepatic EZH2 mRNA levels in rats with NAFLD were evaluated. As shown in Figure 1c, EZH2 transcripts decrease as the disease increases in severity.

However, the expression levels of the EZH2 protein in the liver remained unaltered for all the dietary regimens (Figure 1d), while the expression of H3K27me3 becomes significantly down-regulated in the HFa and HFa/HFr-D rats compared with the SD ones. Furthermore, immunohistochemical staining demonstrated that nuclear positivity for EZH2 was drastically reduced in the livers from both HFa (3.8 ± 5.6) and HFa/HFr-D (1.6 ± 3.7) rats with respect to control (17.8 ± 7.1) as shown in (Figure 1e).

As the uncoupling between the EZH2 mRNA decreases, the unchanged protein levels might reflect the activation of the regulatory pathways/feedback loops that need to be investigated in the future. These data highlight that NAFLD in the *in vivo* models is characterized by a progressive intra-hepatic reduction of nuclear EZH2, commensurate to the down-regulation of H3K27me3 levels.

**Figure 2.**
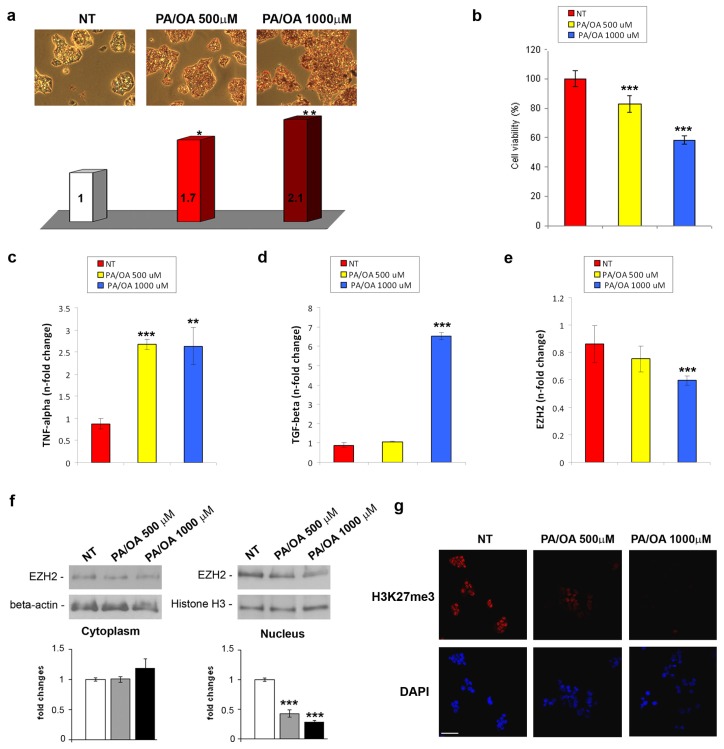
(**a**) (**upper panel**) Representative photomicrographs (20×) and (**lower panel**) spectrophotometric quantification of lipid accumulation by Oil-Red-O in HepG2 cells either normal or treated with 500 or 1000 μM PA/OA for 24 h. Data were expressed as fold changes (at least three independent experimental determinations in quadruplicate) with respect to the control (NT). *p < 0.05, **p < 0.01 *vs*. the control cells; (**b**) Cytotoxic effect of FFAs on HepG2 cells was measured by Cell Proliferation Kit II (XTT). Data, referring to two independent tests, were normalized as % of untreated cells. ***p < 0.001 *vs*. control cells; TNF-α (**c**), TGF-β (**d**) and EZH2 (**e**) mRNA expression levels in FFA-treated or untreated HepG2 cells. The histograms represent the mean ± SD of at least two independent experiments in triplicate. **p < 0.01 and ***p < 0.001 *vs*. the control cells; (**f**) Representative western blotting and quantitative analysis of cytoplasmic and nuclear EZH2. β-actin and histone H3 were used as loading controls. Quantitative bars and the correspondent error bars (SD) are referred to the mean of at least two determinations in three independent experiments. ***p < 0.001 *vs*. SD; (**g**) (**upper panel**) Immunofluorescence of H3K27me3 after 24 h FFA-treatment, compared with non-treated cells (NT). (**lower panel**) DAPI was used to stain nuclei (Magnification 40×). Scale bar, 30 μm.

Next, we established a well-described model of *in vitro* NAFLD by treating the HepG2 cells with different concentrations of palmitic acid (PA) and oleic acid (OA) for 24 h. This treatment mimics the free fatty acids (FFA) lipotoxicity and steatosis which occurs during the course of the disease [26]. As demonstrated by Oil-Red-O staining in Figure 2a, PA/OA induced a statistically significant dose-dependent intracellular lipid accumulation, while a relevant reduction of cell viability, particularly after treatment with 1000 μM PA/OA, was observed (Figure 2b). Furthermore, the FFA treatment, in particular the highest dose (1000 μM PA/OA), induced an increased expression of the TNF-α and TGF-β mRNA (Figure 2c,d).

In order to investigate whether the lipotoxicity of the PA/OA treatments affects the EZH2 expression/activity, we analysed the EZH2 transcriptional levels in the total extracts and its protein levels in both the cytoplasmic and nuclear fractions. We found that the EZH2 mRNA was significantly reduced only in 1000 μM PA/OA treated cells compared with the untreated ones (Figure 2e), whereas the decrease in nuclear protein appeared to be dose-related (Figure 2f, lower panel). Interestingly, the FFA-dependent EZH2 nuclear decrease was not associated with a concomitant cytoplasmic increase (Figure 2f, upper panel), suggesting that the EZH2 protein was down-regulated. Interestingly, immunofluorescent analysis demonstrated a reduction of nuclear expression of H3K27me3 upon stimulation with FFA (Figure 2g).

Collectively, the *in vivo* and *in vitro* findings indicate a significant progressive dose-dependent reduction in the EZH2 nuclear protein levels, which results in the loss of the histone H3 Lys27 trimethylation in NAFLD. The loss of nuclear EZH2 could concur to diet-induced liver damage (*i.e.*, steatosis, ballooning, *etc*.) and to enhanced production-release of TNF-α and TGF-β, considered two of the most relevant hepatic-induced pro-inflammatory molecules in NAFLD progression [27].

These results are in agreement with reports showing the EZH2 protein associated with the Mallory-Denk Bodies and ballooned hepatocytes, both correlated with liver damage, in alcoholic hepatitis [28].

### 2.2. Effects of DZNep on Lipid Accumulation and Hepatic Expression of TNF-α and TGF-β in the Fatty HepG2 Cells

To investigate whether EZH2 was involved in lipid accumulation and inflammation occurring in HepG2 resulting from PA/OA treatments, we treated the HepG2 cells with a prototype of the EZH2 inhibitor, 3-Deazaneplanocin A (DZNep), which acts both on the activity and the stability of the EZH2 protein, through mechanisms that still remain unclear [29,30].

**Figure 3.**
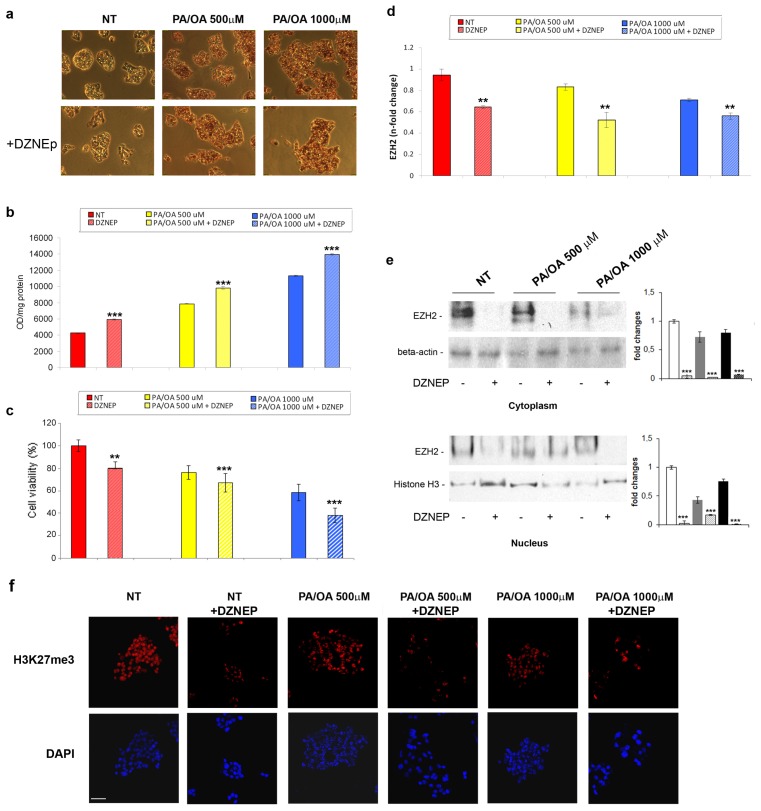
(**a**) Representative photomicrographs (200×) and (**b**) spectrophotometric quantification, expressed as optical density (OD)/mg protein of lipid accumulation by Oil-Red-O in the normal HepG2 cells or those treated with 500 or 1000 μM PA/OA for 24 h post DZNep pre-treatment. The histograms represent the mean ± SD of at least three independent experiments in triplicate. ***p < 0.001 *vs*. respective controls; (**c**) Cytotoxic effect of FFAs and/or DZNep on HepG2 cells was measured by Cell Proliferation Kit II (XTT). Data, referring to two independent tests, were normalized as % of untreated cells. **p < 0.01 and ***p < 0.001 *vs*. control cells; (**d**) EZH2 mRNA expression levels and (**e**) representative western blotting of the total EZH2 protein levels in the FFA-treated or untreated HepG2 cells pre-exposed to DZNep. The histograms represent the mean ± SD of at least two independent experiments in triplicate. **p < 0.01 and ***p < 0.001 *vs*. the respective controls; and (**f**) (**upper panel**) Immunofluorescence of H3K27me3 after previously described treatments, compared with non-treated cells (NT). (**lower panel**) DAPI was used to stain nuclei (Magnification 40×). Scale bar, 30 μm.

As indicated in Figure 3a,b, the treatment with 5 μM DZNep induced *per se* a statistically significant increase in the intracellular lipids in the control cells, and a further augmentation of the PA/OA-dependent lipid accumulation. Of note, DZNep showed additional cytotoxic effects, as measured by XTT, on either the untreated or FFA-treated HepG2 cells (Figure 3c). These data suggest that the DZNep may exert a lipogenic activity inducing a fatty phenotype in the HepG2 cells. These data are in agreement with the ability of DZNep to induce the accumulation of lipid droplets in breast cancer cell lines [29].

Real-Time RT-qPCR and western blotting were performed to determine the DZNep effects on the EZH2 mRNA and protein levels. As reported in Figure 3d,e, the DZNep treatment caused a significant down-regulation of the EZH2 both at the transcript and protein levels in the control, as well as in the FFA-treated HepG2 cells. Interestingly, DZNep induced a reduction of nuclear H3K27me3 that was not increased by the DZNep/FFAs combined treatment (Figure 3f).

**Figure 4.**
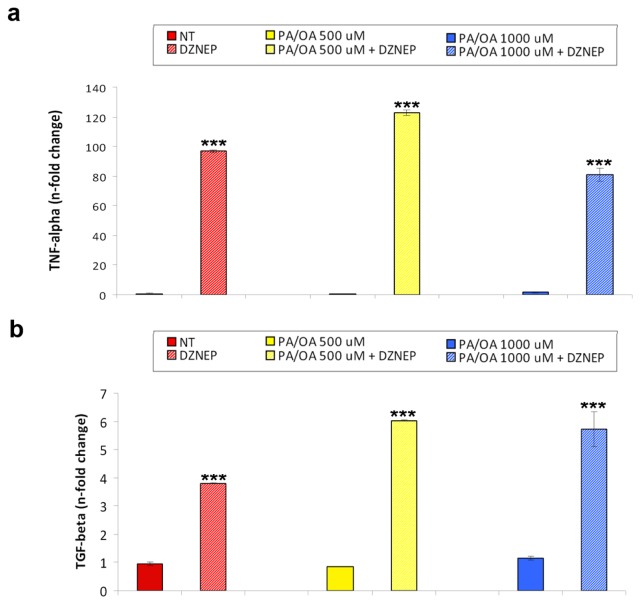
(**a**) TNF-α and (**b**) TGF-β mRNA expression levels in the HepG2 cells either normal or treated with 500 or 1000 μM PA/OA for 24 h after pre-treatment with DZNep. The histograms represent the mean ± SD of at least two independent experiments in triplicate. ***p < 0.001 *vs*. the respective controls.

Next, we evaluated the DZNep effects on FFA-induced hepatic expression of pro-inflammatory gene transcripts. We found that the DZNep caused a significant additive increase in the TNF-α and TGF-β mRNA levels compared with that induced by the PA/OA treatment (Figure 4a,b).

Altogether, these findings suggest that the DZNep-mediated reduction of the EZH2 expression/activity could render the hepatocytes more prone to steatosis development and, in turn, the resultant lipotoxic environment could increase the liver susceptibility to inflammation.

Interestingly, Zeybel *et al*. [31] demonstrated the efficacy of DZNep as an anti-inflammatory and anti-fibrotic agent in mice on a methionine and choline deficient diet, a model for hepatic fibrosis. It is conceivable that the EZH2 function could be diverse in the different stages of NAFLD-related damage. Therefore, the potential ability of DZNep as a candidate for the treatment of NAFLD deserves further investigation in the future.

### 2.3. Effects of DZNep on the Hepatic Expression of miR-200b and miR-155

**Figure 5.**
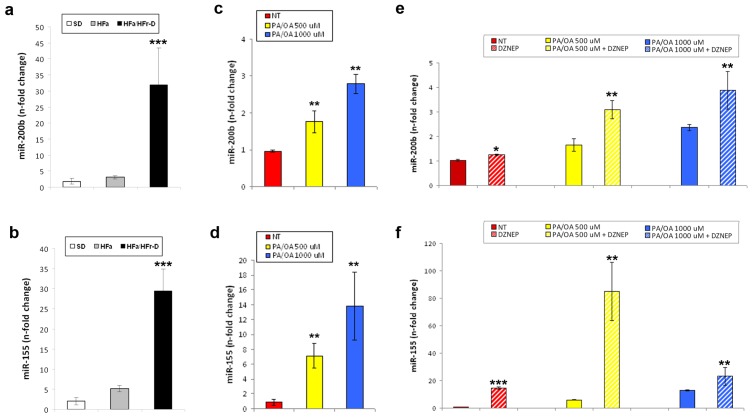
miR-200b (**a**) and miR-155 (**b**) expression levels in SD and HFa/HFr-D rats. Real-Time RT-qPCR results for miR-200b (**c**) and miR-155 (**d**) in HepG2 cells normal or treated with 500 or 1000 μM PA/OA for 24 h; Expression levels of miR-200b (**e**) and miR-155 (**f**) in FFA-treated or untreated HepG2 cells pre-exposed to DZNep. Histograms represent the mean ± SD of at least two independent experiments in triplicate. *p < 0.05, **p < 0.01 and ***p < 0.001 *vs*. the respective controls.

A myriad of mechanisms, still undiscovered, could explain the steatotic and pro-inflammatory effects of EZH2 depletion in the HepG2 cells [32]. Interestingly, Au *et al*., [18] recently demonstrated that the EZH2 is up-regulated in Hepatocellular Carcinoma (HCC) and that its expression is associated with a more aggressive and metastatic phenotype. Moreover, the same authors have found that EZH2 silencing in the HCC cells reduces their metastatic potential by regulating the tumour suppressor miRNAs, such as miR-200b. The up-regulation of miR-200b, as well as of other miRNAs, including miR-155, is correlated with the development and progression of methyl-deficient diet-triggered NAFLD in mice [33]. In our NAFLD rat model, we confirmed the prior data showing the over-expression of miR-200b in the HFa/HFr-D rats when compared with the SD controls (Figure 5a) [34]. Besides, as a new finding, the miR-155 was also observed to be up-regulated (Figure 5b).

Of significance, the transcription of both the miRNAs was greatly enhanced by increasing the PA/OA concentration (Figure 5c,d). Finally, preventive treatments with DZNep triggered a further increase in the expression of the FFA-dependent miRNAs in the HepG2 cells (Figure 5e,f).

Altogether, these results support the hypothesis that EZH2 depletion could partially affect the hepatocyte metabolism and inflammatory response through the regulation of specific miRNAs and their target networks, as demonstrated earlier in methyl deficient-dependent NAFLD [33]. However, further experiments are required to investigate the potential mechanisms that could link EZH2, miR-200b/miR-155 expression, with *in vitro* and *in vivo* NAFLD.

## 3. Experimental Section

### 3.1. Animal Treatments and Histology

As described earlier on [25], the Sprague-Dawley rats (120–140 g), obtained from Harlan Italy (San Pietro al Natisone, Udine, Italy), were fed on three different types of diet: standard diet (SD), a high-fat diet (HFa) (58% of energy derived from fat, 18% from protein and 24% from carbohydrates) and HFa-enriched with 30% fructose in the drinking water (HFa/HFr-D). After 15 weeks, the liver tissues and blood samples were subjected to biochemical, histological, and molecular analyses. The animals received treatment concurring with the European guidelines of the local committee for animal care and welfare and based on the experimental protocol approved by the Italian Ministry of Health.

### 3.2. Histology, Immunohistochemistry and Immunofluorescence

The liver was fixed in 4% buffered formalin and embedded in paraffin. Sections of 3–5 μm thickness were stained with H-E (Bio-Optica, Milan, Italy) or by using antibodies specific for anti-EZH2 (612666; BD Transduction Laboratories, Franklin Lakes, NJ, USA). Detection of the primary antibody was performed by using the appropriate secondary biotinylated antibody (Vector Laboratories, Bridgeport, NJ, USA) and the peroxidase DAB kit (Dako, Carpinteria, CA, USA). Then the specimens to be studied for histology were evaluated under the 10 × 40 light microscopic fields, while those for immunohistochemistry were counterstained with 4′,6-diamidino-2-phenylindole and evaluated using the Leica Microsystems (DM 4500 B, Weltzlar, Germany). The percentage of EZH2-positive nuclei was counted in at least 10 fields at magnification 400× per sample by two blinded independent observers.

For immunofluorescent detection of H3K27me3, HepG2 cells were seeded at 2 × 10^4^ cells/well and grown in a 4-well chamber slide (Nunc, Naperville, IL, USA). After previously described treatments, cells were fixed in a methanol/acetone (2:1) solution at −20 °C for 10 min, and then were incubated overnight with a rabbit anti-H3K27me3 antibody (1:800) purchased from Cell Signaling (9756, Cell Signaling Technology, Inc., Danvers, MA, USA). After three washes in PBS, cells were incubated for 1 h with the secondary antibody Alexa Fluor^®^ 555 Goat Anti-Rabbit IgG (1:100) (Applied Biosystems, Life Technologies, Carlsbad, CA, USA). Nuclei were counterstained with DAPI for 5 min after extensive washing, the samples were mounted with PBS/glycerol (1:1) and covered with a coverslip. Images were acquired by confocal laser fluorescence microscopy with Olympus fluoview FV1000 confocal microscope (Olympus Corporation, Tokyo, Japan) (Magnification 40×).

### 3.3. ELISA

The ELISA-based kits were used to assay the plasma levels of TNF-α (Peprotech, Rocky Hill, NJ, USA) and TGF-β (Biovendor, Brno, Czech Republic).

### 3.4. Cell Lines and Treatments

The HepG2 cell line, purchased from ATCC, American Type Culture Collection (Rockville, MD, USA) was grown, as a monolayer, in Dulbecco’s modified Eagle’s medium (DMEM) supplemented with 10% fetal bovine serum, 2 mM l-Glutamine, 10,000 U/mL penicillin and 10 mg/mL streptomycin at 37 °C under 5% CO_2_ in a 95% humidified atmosphere. To resemble NAFLD condition *in vitro*, the cells were cultured for 24 h in an enriched medium containing increasing concentrations (500 and 1000 μM) of palmitic acid (PA) and oleic acid (OA) in a 1:2 molar ratio. For pharmacological treatments, the HepG2 cells were treated with 5 μM Deazaneplanocin A (DZNep) for 72 h, followed by PA/OA treatment for 24 h.

### 3.5. Oil-Red-O Staining

HepG2 cells were seeded in a 6-wells plate at a density of 1 × 10^5^ cell/well. After treatment with FFA for 24 h or with 5 μM DZNep for 72 h, followed by 24 h-FFA-treatment, Oil-Red-O staining was performed to assess intracellular lipid content. Oil-Red-O (Sigma O-0625, Sigma-Aldrich, St. Louis, MO, USA), a fat-soluble diazo dye, with a maximum absorption at 518 nm, stains neutral lipids. After treatment, the cells were washed twice with PBS and then fixed with 10% formaldehyde for at least one hour. Once the formaldehyde was removed, 60% isopropanol was added to each well and allowed to dry. The intracellular lipids were stained with a 60% dye solution, for 10 min. Images were acquired under an inverted microscope (20×, Nikon Eclipse TE200, Nikon Corporation, Tokyo, Japan).

The intracellular dye content, proportional to the fatty acid accumulation was then determined by measuring the absorption at 500 nm using an ELISA microplate spectrophotometer (ELISA Benchmark Plus, BIORAD, Segrate, Italy), after eluting Oil-Red-O by adding 1 mL of 100% isopropanol for 10 min to each well. All the staining procedures were performed at room temperature.

The results were expressed as optical density (OD)/mg protein of lipid accumulation.

### 3.6. Cell Viability Assays

The cytotoxic effects of FFA and/or DZNep on HepG2 cell survival were evaluated using the Cell Proliferation Kit II (XTT, Roche Molecular Biochemicals, Indianapolis, IN, USA), according to the manufacturer’s protocol. Two independent tests were conducted.

Into each well of a 96-well microtiter cell culture plate, 3 × 10^4^ HepG2 cells were seeded, and, 24 h after plating, cells were treated with FFA (500 and 1000 μM) for 24 h or DZNep for 72 h followed by 24 h-FFA-treatment. The assay was performed in quintuple.

At the end of the treatment, 50 μL of a mixture of XTT labelling reagent and electron-coupling reagent were added and incubated at 37 °C for 4 h. The absorbance of the water-soluble formazan formed was measured at 450 and 650 nm using an ELISA microplate spectrophotomer (ELISA Benchmark Plus, BIORAD, Segrate, Italy) and it is directly proportional to the number of living cells in the culture. The viability of the treated cells was expressed as the percentage of viable cells in relation to untreated cells.

### 3.7. Real-Time RT-qPCR

Total RNA was extracted using TRizol (Invitrogen, Life Technologies, Carlsbad, CA, USA) according to the manufacturer’s protocol and inspected by agarose gel electrophoresis. Reverse transcription was performed using the Improm-II Reverse Transcription System (Promega, Madison, WI, USA). The expression levels were measured by Real-Time RT-qPCR for the relative quantification of the gene expression as described [35]. TaqMan gene assay (Applied Biosystems, Life Technologies, Carlsbad, CA, USA) for EZH2 (Hs01016789_m1) and TNF-α (Hs00174128_m1) were used. The TaqMan probes for human TGF-β and rat EZH2 were supplied by IDT (Integrated DNA Technologies, Coralville, IA, USA, 112396184, 115543267). The samples were normalized according to the glyceraldehyde-3-phosphate dehydrogenase (GAPDH) mRNA (Hs99999905_m1) levels.

Reverse transcription for miRNAs was performed using the TaqMan MicroRNA Reverse Transcription Kit with specific miRNA primers (Applied Biosystems, Life Technologies, Carlsbad, CA, USA). TaqMan microRNA assays (Applied Biosystems, Life Technologies, Carlsbad, CA, USA) were used for relative quantification of the mature miR-200b (hsa-miR-200b; 002251) and miR-155 (hsa-miR-155; 000479) expression levels, as described [36]. U6 snRNA (001973) was used to normalize the results. An Applied Biosystems 7900HT Fast Real-Time PCR System (Applied Biosystems, Life Technologies, Carlsbad, CA, USA) was used for measurements.

The expression fold change was calculated by the 2^−ΔΔCt^ method for each of the reference genes [37]. At least two independent amplifications were performed for each probe, with triplicate samples.

### 3.8. Western Blotting

Total protein extraction was performed by homogenizing tissues and cells in Ripa lysis buffer (50 mM Tris pH 7.5, 150 mM NaCl, 1% Triton X-100, 1 mM EGTA, 1% sodium deoxycholate and phosphatases 1% cocktail protease inhibitors, 0.5 mM sodium orthovanadate) and incubated on ice for 30 min. The homogenates were then centrifuged at 13,000 rpm for 10 min and the resulting supernatant was taken as a protein sample.

The nuclear extracts and cytoplasmic fractions were first separated by resuspending the cellular pellets in a cytoplasmic lysis buffer A (containing 20 mM Hepes pH 7.9, 10 mM KCl, 0.2 mM EDTA, 1 mM DTT, 0.5 mM PMSF, 0.6% NP40) and then centrifuged at 3000 rpm for 10 min The supernatants were equivalent to the cytoplasmic extracts. The pellets containing the nuclei were washed with Buffer A without NP40 and resuspended in Buffer B (containing 20 mM Hepes pH 7.9, 0.4 M NaCl, 2 mM EDTA, 1 mM DTT and 1 mM PMSF). After 30 min of incubation at +4 °C, the pellets were centrifuged at 13,000 rpm for 30 min, and the supernatant, equivalent to the nuclear fractions, was collected. The samples were then diluted in the sample buffer (200 mM Tris-HCl (pH 6.8), 40% glycerol, 20% β-mercaptoethanol, 4% sodium dodecyl sulphate, and bromophenol blue) and resolved in 7.5% and 12.5% SDS-PAGE, then transferred and immobilized onto the nitrocellulose membranes (Amersham, Germany). The membranes were blocked using 5% non-fat dry milk for 30 min and incubated with the appropriate primary and secondary antibodies. The anti-β-actin and the anti-histone3 primary antibodies were supplied by Santa Cruz Biotech (Santa Cruz, CA, USA); anti-EZH2 (612666) and the anti-H3K27me3 (Lys27; 9733) were purchased from BD Biosciences (BD Transduction Laboratories, Franklin Lakes, NJ, USA) and Cell Signaling Technology (Cell Signaling Technology, Inc., Danvers, MA, USA), respectively. Western blotting was performed at least in duplicate for three independent experiments.

### 3.9. Statistical Analysis

The data were presented as the means ± SD. Comparisons were made between the means from at least two independent experiments repeated in triplicate. The statistical differences were analysed using Student *t* test. *p* values < 0.05 were considered statistically significant.

## 4. Conclusions

The disruption of the adaptive mechanism of the epigenetic control by hypercaloric diets may alter the gene expression leading to the development of a wide range of disorders, including NAFLD [7,8]. In this study, we demonstrate that the NAFLD is associated *in vivo* with nuclear EZH2 loss, which is reflected by an *in vitro* model of NAFLD in the HepG2 cells. HepG2 cell treatment with the prototype inhibitor of EZH2, DZNep, demonstrates for the first time that exacerbating the down-regulation of the EZH2 protein and mRNA levels causes the hepatocytes to become more susceptible to lipid accumulation and inflammation. The pro-inflammatory and pro-steatotic effects of DZNep could be dependent on a direct up-regulation of the proteins and/or miRNAs under the control of EZH2, and/or by the action of this drug on other proteins of the PRC2 core, such as EED and SUZ12 [38,39]. These possibilities warrant greater explorative study in the future.

In conclusion, our results suggest that the down-regulation of the EZH2 or a dysfunctional mutated EZH2 associated with gene polymorphisms could be correlated with the high risk of NAFLD developing in humans. Significantly, from a translational point of view, because DZNep is a potential and promising drug useful in the treatment of various types of cancer, the patients who will be eventually treated with DZNep should be monitored for the induction of NAFLD as a potential side effect.

However, a thorough investigation into the potential roles of the EZH2 in the NAFLD pathogenesis is just a preliminary observation that needs to be confirmed and validated. Along these lines, further experiments on *in vivo* transgenic mice could be useful to understand whether the presence of a functional EZH2 is crucial to protect the liver from diet-induced NAFLD, as well as to develop potential targeted-preventive strategies.
